# Liver transplantation in elderly patients: what do we know at the beginning of 2020?

**DOI:** 10.1007/s00595-020-01996-7

**Published:** 2020-04-11

**Authors:** Shimon Dolnikov, René Adam, Daniel Cherqui, Marc Antoine Allard

**Affiliations:** Centre Hépatobiliaire, Paul Brousse Hospital, 14 avenue Paul Vaillant Couturier, 94800 Villejuif, France

**Keywords:** Liver transplantation, Elderly patients, Frailty

## Abstract

An aging population has prompted us to evaluate the indications of liver transplantation (LT) in elderly patients more frequently. In this review, we summarize the short- and long-term results after LT in elderly patients and also discuss the criteria used to select patients and how recipient age can challenge current allocation systems. Briefly, the feasibility and early outcomes of LT in elderly patients compare favorably with those of younger patients. Although long-term survival is less than satisfactory, large-scale studies show that the transplant survival benefit is similar for elderly and younger patients. Therefore, age alone does not contraindicate LT; however, screening for cardiopulmonary comorbidities, and asymptomatic malignancies, evaluating nutritional status, and frailty, is crucial to ensure optimal results and avoid futile transplantation.

## Introduction

Since the first successful liver transplantation (LT) in humans in 1967 [1], this procedure has shifted from a “last chance” treatment to a well-standardized procedure, now accepted as the only curative option for patients with end-stage liver disease. The medical and surgical complexity of LT, combined with the current organ shortage, has led physicians to propose several selection criteria, among which the age of the recipient has always been considered important. In fact, during the 1980s, the recipient age could not exceed 45–50 years [2], whereas now, arbitrary age limits have been abandoned. Transplant teams have become faced with increasing demand among elderly patients, in line with an aging population and also higher incidences of hepatocellular carcinoma (HCC) and non-alcoholic steatohepatitis (NASH) with age [3]. Consequently, several questions related to elderly recipients are emerging. Numerous studies from western and eastern countries have explored the impact of recipient age on post-transplant survival. However, their results are contradictory and the selection policies with regard to the age of the recipient are subject to variations from one center to another. This review discusses the main questions related to LT, excluding living donor LT, in elderly patients, based on the available literature.

## Feasibility of LT in the elderly patients

In 1991, Starzl et. al reported a series of 156 patients older than 60 (the oldest being 76) who underwent LT [4]. The 3-year survival rate was 65% and the authors concluded that *“*Advanced Age per se is Not a Contraindication to Liver Transplantation*”* provided that respiratory and cardiovascular function is adequate. Since the definition of elderly recipients is unclear, the cutoff age in series moved progressively to 65 years and, more recently, to 70 years [5–10]. Data from the United Network for Organ Sharing and the European Liver Transplant Registry show an increase in the proportion of elderly LT recipients over 65 years and over 70 years in the past decade. For example, in the United States, the proportion of registrants aged ≥ 65 years rose from 8% in 2002 to 17% in 2014 [11]. The same trend was observed in recipients aged ≥ 70 years (1.4–3.1%). In 2010, a publication based on UNOS data revealed that four patients ≥ 80 years old were transplanted [12]. These results support the feasibility of LT in elderly patients is now widely agreed. Table 1 summarizes the findings of several series showing the feasibility of LT in septuagenarians.

**Table 1:** Overview of liver transplant results in septuagenarians

References	Year	Study population	Donor type	No. of elderly patients	Selection criteria	Early outcomes	Long-term outcomes
Rudich [63]	1999	Single center, USA	DDLT	33 (> 70 yrs)	N/A	Similar complication rates	1-year OS: 60%
Aduen [7]	2004	Single center, USA	DDLT	42 (≥ 70 yrs)	Cardiac stress testing and colonoscopy	Similar complication rates	1-year graft loss: 17% but 5-year OS: 63%
Safdar [64]	2004	Single center, USA	DDLT	33 (≥ 70 yrs)	N/A	No comparison	1-year OS: 78%; 3-year OS: 71%
Lipshutz [6]	2007	Single center, USA	DDLT	62 (≥ 70 yrs)	All patients underwent cardiac workup, regardless of age	Similar complication rates	1-year 73%; 5-year OS: 47%
Schwartz [5]	2012	UNOS Registry	DDLT (only 1 LDLT)	743 (≥ 70 yrs)	N/A	N/A	1-year actuarial OS: 81%; 5-year actuarial OS: 55%
Taner [9]	2012	Single center, USA	DDLT	13 (≥ 75 yrs)	Cardiac stress echo, pulmonary function, nutritional assessment	No comparison	1-year OS: 100%; 5-year OS: 50%
Lai [48]	2014	UNOS Registry	DDLT	343 (≥ 70 yrs)	N/A	No comparison	1-year graft survival was 56% in patients with MELD ≥ 28 vs. 82% in recipient with lower MELD score
Wilson [8]	2014	SRTR and UHC databases, USA	DDLT	323 (≥ 70 yrs)	N/A	Similar complication rates	1-year OS: 85%; 5-year OS: 64%
Oezselik [65]	2015	Single center, Turkey	LDLT	12 (> 70 yrs)	N/A	No comparison	2 deaths within 6 months post-LT (17%)
Kwon [10]	2017	Single center, Korea	LDLT–DDLT	25 (> 70 yrs)	Echocardiography, coronary CT angiography, thallium scan of myocardial perfusion, pulmonary function test, brain MRA	Similar complication rates	1-year OS: 84%; 5-year OS: 70%
Sharma [66]	2017	UNOS Registry	DDLT	1511 (≥ 70 yrs)	N/A	No comparison	5-year OS about 60%
Gil [67]	2018	KNHI, Korea	LDLT–DDLT	84 (> 70 yrs)	N/A	Higher early mortality rates	20% hospital mortality
Mousa [68]	2019	Single center, USA	DDLT	162 (≥ 70 yrs)	N/A	Similar early survival rates	5-year OS: 71%

*NA* Not available, *DDLT* deceased donor liver transplant, *LDLT* living donor liver transplantation, *USA* United States of America, *UNOS* United Network for Organ Sharing, *SRTR* Scientific Registry of Transplant Recipients, *UHC* University Health System Consortium, *MRA* magnetic resonance angiography, *KNHI* Korean National Health Insurance, *OS* overall survival

## Safety of LT in elderly patients

Studies investigating the factors impacting early survival after LT have identified two categories of predictors: those related to recipient conditions such as sarcopenia, MELD score, and organ failure at the time of transplant [13–15] and those related to post-transplant function recovery of the graft [16–18]. The practical consequence of these findings is that optimal grafts should be given to the sickest recipient. Notably, the age of the recipient did not clearly predict early survival. Moreover, studies that compared the early outcomes of young vs. elderly recipients did not report higher rates of mortality, or vascular or biliary morbidity [7, 19–23]. One of three available studies [20, 22, 23] reported a higher incidence of neuropsychiatric complications in the elderly group. The recent meta-analysis of Gomez Gavara did not find any difference in the risk of complications between young and elderly recipients [24]. It could be argued that the absence of the impact of age on early outcomes could result from a stringent selection of elderly recipients. Indeed, despite that the prevalence of cardiovascular comorbidities and diabetes increases with aging, not all studies reviewed in this work demonstrated significantly higher proportions of comorbidities in elderly recipients.

## Long-term outcomes after LT in elderly patients

Several scoring systems have been proposed for predicting long-term outcomes after LT. Most of the predictors are related to donor factors, intraoperative data, and parameters of the recipient. The age of the recipient had predictive value in several scores, such as the SOFT, the BAR score, and the donor to recipient model [25–27].

Similarly, data from American and European registries have shown lower post-transplant overall survival of older patients [5, 28]. The actuarial survival at 5 years was 55% for UNOS patients older than 70 years vs. 73% for younger recipients. In Europe, 5-year survival rates were 66% for recipients over 60 years vs. 73% for recipients aged 46–60 years old. The natural lower life expectancy can explain the association between advancing age and increased mortality among old patients. In fact, the differences in life expectancy between younger and older age groups can be accounted for as in cancer research [29]. This approach may also be considered in the field of transplantation. Some investigators have pointed out that analysis based on crude survival is not valid and that we should think in terms of survival benefit from transplantation instead of post-LT survival. This suggests that a specific statistical model should be used [30]. Rather than a Cox model, which gives the hazard ratio of death according to age, eventually adjusted to confounding factors, a transplant-related survival benefit should be used, as it can be defined. Consequently, Su et al. found that advanced age among UNOS transplanted patients had no impact on the transplant-related survival benefit for equivalent MELD scores or among patients with vs. those without HCC [11]. This finding was explained by the fact that although post-LT survival was lower in older patients, the risk of death or exclusion from a waitlist, because of deterioration of general status, was higher. They concluded that patient age alone should not be used to disqualify a potential candidate for LT and that the current aging of recipients does not impair the survival benefit obtained by transplantation. Based on this discussion, age may be considered a variable in a potential future score, perhaps as a continuous variable, as suggested by Garcia et al. [31].

## Selection of elderly candidates for LT

As reported previously, LT in elderly patients is feasible and does not seem to be associated with higher surgical risk. Moreover, transplant survival benefit remains similar to that of younger patients. Based on this evidence, the European Association for the Study of the Liver decided that age is not a limitation when considering LT for an elderly patient [32]. Similarly, the American Association for the Study of the Liver 2013 recommends considering physiologic rather than chronological age when evaluating a patient for LT [33]. However, both emphasized the need to screen for comorbidities.

- **Comorbidities and cancer screening**
  Good cardiac function is required to cope with the hemodynamical stress related to LT itself and other potential events such as hemorrhage and reperfusion syndrome. However, cirrhotic patients often suffer so-called cirrhotic cardiomyopathy, combining systolic and diastolic dysfunction, and electrophysiological abnormalities [34]. This pre-existing cardiomyopathy can be worsened by coronary arterial disease (CAD), the prevalence of which increases with aging. It is estimated that 27% of patients over 50 years old with liver disease have moderate to severe coronaropathy [35]. A case—control study did not find an increased prevalence of CAD in patients with cirrhosis. Traditional cardiovascular risk factors remain relevant and should guide preoperative evaluation [36]. Specific modalities of cardiac assessment are not well codified, but there is a consensus to evaluate a candidate for LT by electrocardiogram and transthoracic echocardiography [32]. Patients with risk factors should have a cardiopulmonary exercise test done to diagnose asymptomatic CAD. This test also measures aerobic capacity, which is predictive of post-LT outcomes [37]. Although initial studies have reported that CAD carries high-risk post-transplant morbidity and mortality [38, 39], a recent multicentric study found a similar post-LT survival for patients with obstructive CAD and patients without obstructive CAD, provided they received adequate treatment prior to transplant. Specifically, age > 55 years with CAD was not found to be associated with higher mortality [40].
  Screening for asymptomatic malignancies is mandatory before enrolling a patient. For patients older than 50 years, colonoscopy is recommended to detect colorectal cancer. In patients considered at risk, CT colonography can be an alternative. For patients with alcoholism or smokers, workup should rule out neoplasia arising from the lung, ear–nose–throat region, bladder, and esophagus. Chest CT scan, consultation with an ENT specialist and a stomatologist, and upper GI endoscopy (ideally during the same session with colonoscopy) are also useful. Dedicated consultation with a gynecologist and a dermatologist is also recommended [32].
- **Nutritional status**
  Nutritional status is a key factor to ensuring success after LT in all patients, irrespective of age. According to one study, recipients with a BMI < 18.5 kg/m^2^ had the worst outcomes [41]. Since advanced age is a risk factor for malnutrition, physicians should pay attention to the nutritional status of older recipients. This evaluation is complex because the usual measures of assessing nutritional status are of little value in the setting of end-stage liver disease. The best surrogate marker seems to be sarcopenia, quickly evaluated by measuring the thickness of the psoas muscle on CT scan. Sarcopenia was demonstrated to be a strong predictor of post-LT mortality [13]. The 3-year survival rates ranged from 26 to 77% for the lowest to the highest quartiles of the psoas area, respectively. If poor nutritional status is a certain risk factor for early mortality, correcting malnutrition remains, in practice, an elusive goal for patients in poor general health [42]. No clear strategy to treat malnutrition in patients with cirrhosis has been established. Some groups have proposed placing an enteral feeding tube before LT in severely malnourished patients and a feeding jejunostomy during LT [43, 44].
- **Frailty index**
  Frailty is a condition which embodies weakness, muscle wasting, exhaustion, slow walking, and limited activity. Its prevalence is high in older adults. The presence of three or more of the above criteria defines frailty, according to Fried [45]. The 6-min walk distance test was used initially to assess the relevance of frailty in LT candidates. Commonly used in patients with cardiac or pulmonary disease, this simple test was an efficient predictor of mortality after adjusting to confounding factors [46]. Frailty was observed in 43% of patients with end-stage liver disease and was also associated with a higher risk of depression [47]. Lai et al. tested the concept of frailty in candidates awaiting LT and found that the risk of mortality or delisting increased by 45% per each point increase of the frailty index [48]. The negative impact of frailty on waiting list mortality has also been confirmed, but this effect remains unmodified by age [49]. Similar findings in patients ≥ 65 years were reported using a different method for assessing frailty, a short physical performance battery [50].
  In elderly patients, candidacy for LT should be evaluated in light of sarcopenia, frailty, and cardiopulmonary reserves, which can be assessed easily by these methods. Combining this information is necessary to recognize which older patients are poor candidates for LT and which older patients are good candidates despite their age. These points also emphasize the need for a geriatric evaluation when older patients are referred for LT.
- **Severity of liver disease and age**

Should we take the severity of the liver disease itself into consideration when considering elderly transplant patients? As early as 2001, Levy et al. reported poor survival after LT in older patients at high risk, namely those hospitalized in an intensive care unit at the time of transplant or with high serum bilirubin levels [51]. Since 2002, the MELD score has become a worldwide predictor of mortality for patients waiting for a LT. Interestingly, the lower MELD scores of older recipients in most of the studies published during the MELD era [6, 8, 11, 52, 53] suggest that LT was reserved for older candidates with less severe disease and that a form of unconscious or conscious selection was applied. Despite this selection, some studies have reported poor results after transplanting elderly patients with high MELD scores [20, 48]. Similarly, a large analysis of transplanted patients 60 years old or older from the UNOS registry during 1994–2005 found that mechanical ventilation and creatinine were among the other independent predictors of post-transplant survival [12].

## Age and graft allocation systems

Considering LT in the elderly also raises questions about the graft allocation system. An optimal allocation system aims to maximize "utility" while respecting "equity or justice" and avoiding "futility." To address the issue of utility, most of the allocation rules rely on the "sickest first" principle. In a geographic area of severe graft shortage, this approach, mainly based on the MELD score, is considered the most reasonable [54]. Some adjustments are made using waiting time or prioritization so that patients needing LT, but with a low MELD score (cholangitis, cancer, metabolic disorder), have an equal chance for a transplant. However, MELD-based allocation systems carry the risk of futile LT if there is no definite limit in the severity of the patient’s condition beyond which post-LT mortality is too high to justify LT. Countries with high donation rates have not chosen this approach. This more favorable situation allows centers to choose the optimal recipient for a given graft to maximize good long-term results.

Deciding on the most appropriate kind of graft for an elderly patient is important. According to a recent study, models with highest performance in predicting graft survival are those that are dominated by donor factors, suggesting that it is mainly donor-related factors that affect long-term graft survival. In contrast, short-term outcomes are best predicted by models dominated by recipient-related factors [55]. Given that old grafts are associated with lower long-term survival in many scoring systems [25, 26, 56, 57], but with little impact on short-term outcomes, an old graft for older recipients with shorter life expectancy could be an acceptable strategy. If the allocation rule is based only on age matching, this could lead to inequities in the chance of receiving an organ. Since there are more elderly donors than young donors, young recipients would have to wait much longer than older recipients [58]. Cucchetti et al. proposed an “age mapping” approach, working in two steps: first, every patient has an equal chance to obtain a graft, but the best livers (basically, the youngest grafts) should be given to patients with the longest life expectancy. They also demonstrated that giving a graft from an old donor to a young recipient is more detrimental than giving it to an old recipient [59].

An argument against the “older to older” approach is that recipient age + donor age > 120 was found to be the strongest independent predictor of poor survival in the UNOS registry [12]. This would mean that a recipient over 65 years would be given a graft from a donor younger than 55 years, which is impossible in countries with severe organ shortage. Given the other factors contributing to post-LT survival in this study, it seems that “older to older” is feasible provided that the recipient does not suffer from additional organ failure and is "fit" for transplant after meticulous workup. A new allocation system was implemented in the United Kingdom in 2018, in which the guiding principles rely upon the transplant benefit score (TBS) and proportional offering by waiting time [60]. The TBS is defined as the difference between the patient's expected utility from the transplant and their need. These two numbers are calculated by 27 variables, including the ages of the recipient and donor. This illustrates again that the prognostic value of the recipient’s age itself should be analyzed while considering numerous other parameters.

## Conclusion

The age of LT recipients is increasing with the world’s aging adult population. Thus, the indications for LT in the elderly are becoming a frequent focus of discussion. There is growing consensus that LT in the elderly is feasible and has acceptable short- and long-term results, comparable to those of younger adults, and offering similar transplant benefit. However, there is no consensus regarding the optimal patient selection process in this population and the cost-effectiveness of transplanting older patients as opposed to younger patients has not been addressed. Moreover, quality of life, one of the secondary goals of transplantation, has not been sufficiently studied in older recipients [61]. The place of retransplantation in elderly patients also remains unclear. The emotional toll of decisions about whether or not to transplant a given patient as well as the emotional factors affecting these decisions is rarely mentioned in the literature. Previous reviews have pointed out that studies available on this topic are retrospective and make it difficult to draw firm conclusions [24, 62]. Critical factors of successful LT in the elderly have been identified and assessment tools are available. However, the indication for LT in the elderly patient is a complex decision, for which a multidisciplinary approach is a prerequisite.
